# Deformation Time Series and Driving-Force Analysis of Glaciers in the Eastern Tienshan Mountains Using the SBAS InSAR Method

**DOI:** 10.3390/ijerph17082836

**Published:** 2020-04-20

**Authors:** Weibing Du, Weiqian Ji, Linjuan Xu, Shuangting Wang

**Affiliations:** 1 School of Surveying and Land Information Engineering, Henan Polytechnic University, Jiaozuo 454003, China; dwb@hpu.edu.cn (W.D.); weiqian1833919@163.com (W.J.); wst@hpu.edu.cn (S.W.); 2 Key Laboratory of Sediment, Ministry of Water Resources, Yellow River Institute of Hydraulic Research, Yellow River Conservancy Commission, Zhengzhou 450003, China; 3 Department of Hydraulic Engineering, State Key Laboratory of Hydro-Science and Engineering, Tsinghua University, Beijing 100084, China

**Keywords:** glacier change, time series analysis, deformation of LOS, SBAS InSAR

## Abstract

Glacier melting is one of the important causes of glacier morphology change and can provide basic parameters for calculating glacier volume change and glacier mass balance, which, in turn, is important for evaluating water resources. However, it is difficult to obtain large-scale time series of glacier changes due to the cloudy and foggy conditions which are typical of mountain areas. Gravity-measuring satellites and laser altimetry satellites can monitor changes in glacier volume over a wide area, while synthetic-aperture radar satellites can monitoring glacier morphology with a high spatial and temporal resolution. In this article, an interferometric method using a short temporal baseline and a short spatial baseline, called the Small Baseline Subsets (SBAS) Interferometric Synthetic-Aperture Radar (InSAR) method, was used to study the average rate of glacier deformation on Karlik Mountain, in the Eastern Tienshan Mountains, China, by using 19 Sentinel-1A images from November 2017 to December 2018. Thus, a time series analysis of glacier deformation was conducted. It was found that the average glacier deformation in the study region was −11.77 ± 9.73 mm/year, with the observation sites generally moving away from the satellite along the Line of Sight (LOS). Taking the ridge line as the dividing line, it was found that the melting rate of southern slopes was higher than that of northern slopes. According to the perpendicular of the mountain direction, the mountain was divided into an area in the northwest with large glaciers (Area I) and an area in the southeast with small glaciers (Area II). It was found that the melting rate in the southeast area was larger than that in the northwest area. Additionally, through the analysis of temperature and precipitation data, it was found that precipitation played a leading role in glacier deformation in the study region. Through the statistical analysis of the deformation, it was concluded that the absolute value of deformation is large at elevations below 4200 m while the absolute value of the deformation is very small at elevations above 4500 m; the direction of deformation is always away from the satellite along the LOS and the absolute value of glacier deformation decreases with increasing elevation.

## 1. Introduction

Most glaciers in China lie in high mountainous environments [1], which are of great significance to the development of agriculture and animal husbandry in arid or semi-arid areas. The monitoring of glaciers is important for the protection of the ecological environment in desert regions [2]. Moreover, glacier melting affects human societies by changing the seasonal water cycle, increasing the rate of natural hazards, and causing sea-level rise [3]. The accurate estimation of glacier variation is important for the prediction and planning of water resources [4,5]. Glaciers are sensitive to rising air temperature and are therefore among the best natural indicators of climatic change [6]. Glacier morphology change is important basic information for monitoring glacier reserves, glacier thickness change, glacier melting, and glacier mass balance, and is thus of great significance for analyzing the driving and resisting forces of glacier motion [7,8]. Hence, in the context of climate change, glacier morphology change is an important factor for regional economic development and environmental protection, since it provides reliable information for water allocation and the prediction of water availability [3,9,10]. Glacier morphology includes glacier area, glacier surface elevation, slope, thickness, thickness of debris, size and number of ice cliffs, etc., and changes in the glacier terminus play an important role in the variation of glacier morphology [11,12]. Glaciers on Karlik Mountain, can be classified as continental glaciers with weak variations; most of them are covered by clean ice at glacial terminuses. On the contrary, glaciers on Qinghai-Tibet Plateau can be classified as maritime glacier, debris cover are the main features for the glacier tongues [13]. Additionally, based on the glacier boundaries obtained from the second China Glacier Inventory (CGI2), which were delineated from multispectral images [14], most of the glaciers on Karlik Mountain are flat-top glaciers or valley glaciers with clean ice at the terminus. In the present study, deformation information from the Small Baseline Subsets (SBAS) Interferometric Synthetic-Aperture Radar (InSAR) method was used to better reflect the variation of glacier size, glacier surface elevation, and glacier thickness.

The routine monitoring of changes in glacier morphology mainly involves onsite investigation via field operations including field stereophotogrammetry, the Differential Global Positioning System technique, and ground-penetrating radar analysis [15]. Such field measurements can obtain accurate monitoring results; however, due to the vast extent, complicated topography, and extreme weather conditions of glaciers, it is very difficult to obtain data of glacier morphology change [8,16]. Additionally, cloudy weather can significantly reduce the accuracy of mapping based on passive optical images of land cover in alpine regions [16]. Consequently, the regional-scale surveying of land cover is still temporally or spatially limited to airborne visible photogrammetry or spaceborne visible remote sensing images [8,17].

Gravity satellite data, laser altimetry data, and InSAR data have been widely used in the study of glacier morphology [17]. Among these, InSAR can provide data with a higher spatial and temporal resolution. More importantly, InSAR observations are not affected by clouds or rain and can be used for observations at both day and night, which makes it a promising and widely used tool [17,18,19,20]. Data from satellite missions such as Envisat, Radarsat, Advanced Land Observing Satellite (ALOS), Shuttle Radar Topographic Mission (SRTM), TerraSAR, and Sentinel have been effectively used for global land surface terrain monitoring [21]. As an all-weather and high-precision information extraction technology, InSAR has been widely used for the monitoring of changes in glacier velocity and morphology [15,22]. However, if the image pairs used to produce InSAR images have a longer time interval, the decorrelation would cause that it is therefore difficult to obtain deformation time series. In order to overcome this problem, several approaches aimed at conducting time series analysis and improving the accuracy of InSAR-based deformation time series have been presented [23,24,25,26]. One of these methods is SBAS InSAR [25,27], which is an advanced InSAR approach that allows the generation of mean deformation velocity maps and deformation time series from a dataset of SAR (Synthetic Aperture Radar) images with a spatial resolution of centimeters to millimeters [22,25]. Compared with conventional SAR monitoring, SBAS InSAR technology reduces the errors caused by the spatial and temporal decorrelation on interference fringes using the multi-temporal technique [27]. Furthermore, SBAS InSAR can obtain continuous, large-scale, and high-precision deformation information, which plays an important role in the extraction and evaluation of changes in glacier morphology [15]. In recent years, many scholars have used InSAR techniques to monitor land surface deformation. For example, Du et al. [15] used the InSAR technique to calculate the mean vertical deformation velocity of glaciers in southeast Tibet using ALOS-PALSAR data from 2007 to 2010 and showed that the glacier surface elevation in this region decreased by 16 mm/year [15].

Karlik Mountain, located in the easternmost part of the Tienshan Mountains, contains numerous glaciers, which represent a critical water source for river supply and industrial and agricultural development [6,28]. Under the influence of global climate change, glaciers worldwide are generally showing a tendency to retreat, which has a serious impact on agricultural production, human life, and ecological environment in the arid region of Western China where water resources are scarce. Glacier deformation is an important index of glacier movement, which can reflect the general trend of regional glacier change. In previous studies, SBAS InSAR data has been used to measure coast uplift, land subsidence, landslides, permafrost, etc. [29,30], which are very different from glacier deformation. This study attempted to use SBAS InSAR data to quantitatively measure glacier area and study glacier surface movement. In this article, the SBAS InSAR technique was used to calculate the mean deformation velocity and deformation time series of glaciers on Karlik Mountain from a large dataset of Sentinel-1A SAR images spanning the time period from 18 November 2017 to 31 December 2018. Additionally, the relationships between glacier deformation and meteorological factors and altitude are discussed.

## 2. Data and Methods

### 2.1. Study Area

**Figure 1:**
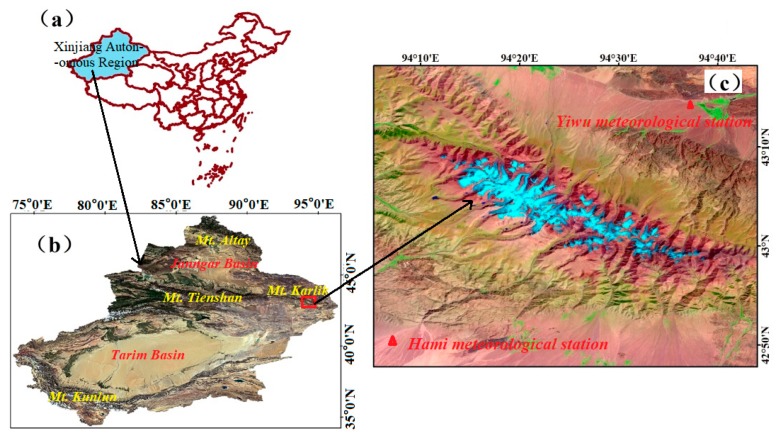
The geographical location of the study area. (**a**) Map of China showing Xinjiang Autonomous Region (XAR). (**b**) Location of Karlik Mountain (red rectangle) within the XAR. (**c**) Landsat 5 image of Karlik Mountain, with glaciers represented by the blue area.

This study focused on Karlik Mountain, located in the Hami region, China (42°50′–43°35′ N, 93°41′–95°07′ E). The mountain is located in the easternmost part of the Tienshan Mountains [15], with an average altitude of about 4000 m (Figure 1). The mountain is high and broad along the ridge line, low and narrow at the sides, forming a dome structure and providing a large accumulation space for glacier development. The eastern, southern, and northern parts of Karlik Mountain are relatively low and surrounded by desert, and the climate is dry in these parts [15]. Data from the Hami meteorological station show that the average annual temperature at the station is −9 °C and the average temperature during June–August is −0.9 °C. As it is located in the extremely dry inland region of Northwest China, the precipitation in the study area is very low; the annual precipitation in the foothills area is less than 100 mm on the southern slopes and less than 200 mm on the northern slopes. However, with increasing altitude, the annual precipitation increases, and can reach 400–500 mm at altitudes of more than 4000 m and more than 600 mm near the glacier equilibrium line.

The glaciers of Karlik Mountain consist of many small flat-top glaciers, valley glaciers, hanging glaciers, and cirque glaciers, with hanging-type glaciers being dominant [30,31]. The mountain is flat, and the summit of the mountain is covered by snow all year round. Additionally, due to the influence of the water vapor effect, the distribution of glaciers is greater in the south of the mountain and lower in the north. According to the first China Glacier Inventory, there are 122 glaciers on the mountain, with a total glacier area of 125.89 km^2^ and a glacier reserve of about 7.01 km^3^. There are 76 rivers in the Hami region, 20 of which are affected by glacial meltwater supply.

### 2.2. Data

Sentinel-1 is an earth observation radar satellite mission launched by the European Space Agency (ESA). Sentinel-1 consists of two satellites, Sentinel-lA and Sentinel-lB, which share the same orbital plane with a 180° difference in orbital phasing. Each satellite has a 12-day revisit frequency, double star cooperation, and the revisit frequency can be shortened to 6 days [22,32]. Sentinel-1 guarantees reduced spatial baselines and short temporal baselines (down to 12 days) and additionally has an orbital tube diameter of about 200 m [22,33], which makes it appropriate for the monitoring of slow deformation. Combined with other satellite data, Sentinel-1 data are widely used in land, ocean, atmospheric, and environmental monitoring, as well as in disaster response and the monitoring of changes to the earth’s surface.

Sentinel-1A was launched on 3 April 2014 with a C-band SAR sensor. Sentinel-1A SAR data are freely and openly available from the ESA’s Sentinel-1 online data hub (https://sentinel.esa.int/web/sentinel/sentinel-data-access/) [34]. The Sentinel-1A satellite has four modes, Interferometric Wide (IW) swath, Extra Wide (EW) swath, Wave Mode (WV), and Strip Map (SM) to produce Single Look Complex (SLC) products [35]. The IW mode includes three sub-swaths, which can enlarge the image swath to 250 km at a higher spatial resolution. For this reason, the IW mode is more suitable for use in this work given the size of the study area. For the C band, which has a wavelength of about 5 cm (i.e., a frequency of about 6 GHz), the Vertical transmit and Vertical receive (VV) polarization mode shows stronger backscattering intensity than the Vertical-Horizontal mode for glaciers, ice, water, etc. [36,37]. The images used in this study are Level-1 SLC products with IW swath and VV polarization mode; the parameters of the experimental data are shown in Table 1 [33,34,35]. The SRTM was a NASA mission which obtained elevation data for most of the world; the mission began on 11 February 2000, and over 222 h and 23 min acquired radar image data covering an area of up to 119 million km^2^ from 56° S to 60° N [35]. The SRTM has two products, a 3” product at a resolution of 90 m and a 1” product at a resolution of 30 m. In this paper, the 3” product was used for terrain phase removal. This data can be acquired from (http://srtm.csi.cgiar.org/srtmdata/) [38]. The DEM data used for this study are SRTM DEM version 4.1 [35], and parameter information for these data is shown in Table 1 [35,39]. Temperature and precipitation data were obtained from the daily surface climate dataset of the National Meteorological Information Center (http://data.cma.cn/).

### 2.3. Methods

SBAS InSAR is a newly developed time series analysis method based on Differential InSAR [40,41,42] which is able to reduce phase noise and error. Additionally, this method is able to isolate the phase that is caused only by glacier deformation [43]. The SBAS InSAR technique is based on the interference of multiple pairs of main images and uses the high-coherence points to recover deformation time series [44]. When choosing the interference pair, it is necessary to consider the distribution of temporal baseline and spatial baseline of the image. The choice of appropriate values of the spatial and temporal baseline threshold can overcome certain spatial and temporal incoherence effects and reduce the impact of noise [45]. Moreover, SBAS InSAR limits the geometric decoherence resulting from long baselines; therefore, during the processing of SAR data, an unlimited amount of SAR interferometric pairs can be utilized to enhance the sampling rate in temporal space [42,46]. In this section, the basic rationale of the SBAS technique is briefly summarized.

Firstly, there are N SAR images covering the research area in a certain time span, appropriate values of spatial temporal baseline thresholds are set, and the baseline combination is conducted according to the principle of small baseline to generate M multi-looked interferometric pairs [47]. M satisfies the following equation [47]:

(1) $$\frac{N+1}{2}<M<\frac{N(N+1)}{2}$$

*M* is determined by the combination of small baselines. An accurate value of M can be determined by setting a spatial baseline threshold and a temporal baseline threshold. In this study, the spatial baseline threshold was set as 2% of the maximum spatial baseline value (unit: m), and the temporal baseline threshold was set as 50% of the maximum temporal baseline value (units: days). This set of threshold values maximally preserves the deformation time series information and effectively reduces data redundancy.

Combined with external Digital Elevation Model (DEM) data (SRTM), M interference pairs were differentially processed by removing the flat-earth and topographic effects. Then, spatial filtering and phase unwrapping were performed to obtain M phases. For one pair of interference pairs, assuming the acquisition time of the main image is $t_{2}^{s}$ and the acquisition time of the slave image is $t_{1}^{m}$, then the phase value of a pixel *i* can be expressed as follows [48]:

(2) $$\Delta \varphi_{i}=\varphi (t_{2}^{s})-\varphi (t_{1}^{m})=\frac{4\pi }{\lambda }\left[d\left(t_{2}^{s}\right)-d\left(t_{1}^{m}\right)\right]+\Delta \varphi_{top}+\Delta \varphi_{atmo}+\Delta \varphi_{noise}$$

where i∈(1,⋯,M), and φ and λ are the interference phase and the radar wavelength, respectively. Assuming that the deformation at $t_{0}$ in this region is zero, $d\left(t_{2}^{s}\right)$ and $d\left(t_{1}^{m}\right)$ are the cumulative variables of the main and slave images, respectively, relative to the direction of Line of Sight (LOS) at time $t_{0}$. $\Delta \varphi_{top}$ is the phase difference caused by the terrain, $\Delta \varphi_{noise}$ is the phase difference caused by the atmospheric effect, and $\Delta \varphi_{noise}$ is the phase difference caused by noise. To make the solved phase continuous, the mean phase change velocity of the image pair j is used, *V*j (j ∈ [1, M]). Then, the M interference phase difference of a pixel *i* can be expressed by Equation (3) [42]:

(3) $$\left[\begin{array}{c} \Delta \varphi_{1}\\ \Delta \varphi_{2}\\ \Delta \varphi_{3}\\ \begin{array}{c} \vdots \\ \Delta \varphi_{j}\\ \vdots \\ \Delta \varphi_{M} \end{array} \end{array}\right]=\left[\begin{array}{ccccccc} \Delta t_{1} & 0 & 0 & 0 & 0 & \cdots & 0\\ 0 & \Delta t_{2} & 0 & 0 & 0 & \cdots & 0\\ 0 & 0 & \Delta t_{3} & 0 & 0 & \cdots & 0\\ \vdots & \vdots & \vdots & \vdots & \vdots & \vdots & \vdots \\ 0 & 0 & 0 & \cdots & \Delta t_{j} & \cdots & 0\\ \vdots & \vdots & \vdots & \vdots & \vdots & \vdots & \vdots \\ 0 & 0 & 0 & 0 & 0 & \cdots & \Delta t_{M} \end{array}\right]•\left[\begin{array}{c} V_{1}\\ V_{2}\\ V_{3}\\ \vdots \\ V_{j}\\ \vdots \\ V_{M} \end{array}\right]$$

where $\Delta \varphi_{j}$ refers to the phase difference of pixel j (j ∈ [1, M]), $\Delta t_{j}$ refers to the time difference of pixel j (j ∈ [1, M]), and *V*j (j ∈ [1, M]) refers to the mean phase change rate of the *V*j (j ∈ [1, M]). Simplified to a vector form, the phase difference of the pixel in the direction of LOS is expressed by the following equation [49]:

(4) $$\Delta \varphi_{LOS}=C•V_{phase}{}^{T}$$

The mean phase change velocity $V_{\mathrm{phase}}$ of the pixel in the adjacent time interval is obtained, and the integral is calculated according to the corresponding time interval. Using the same method, the phase change of the time series of each pixel is obtained, and the final deformation of the study area in the direction of LOS is obtained. When the deformation value is negative it means that the ground feature has moved away from the satellite along the LOS direction, while when the deformation value is positive it means that the ground feature has moved towards the satellite along the LOS direction.

**Figure 2:**
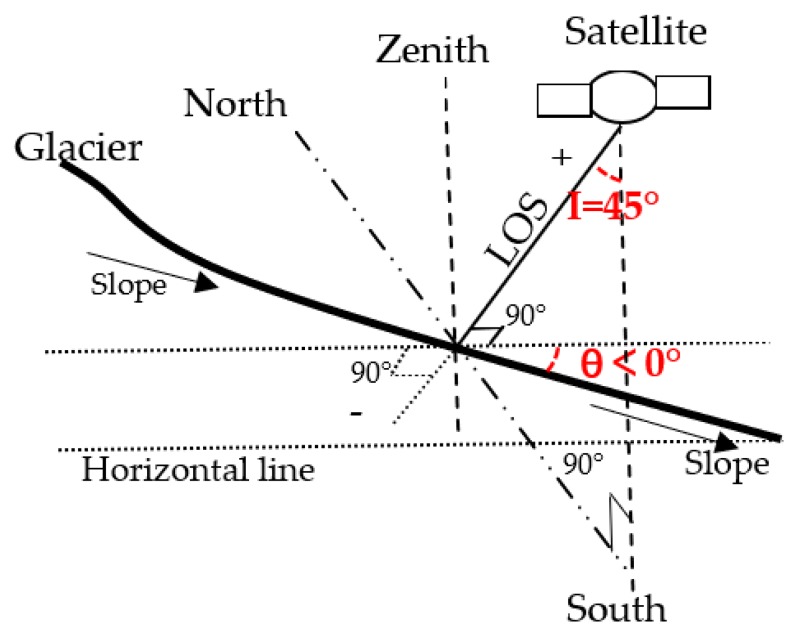
The relative positions of the Line of Sight (LOS), zenith, slope direction, and horizontal line for the profile of the glacier central line. The symbols “+” and “−” refer to positive and negative deformation, respectively. “I” means incidence angle, “θ” means the angle between slope direction and the horizontal line.

Here, the maximum incidence angle of Sentinel-1A is 45°. When the slope is larger than this maximum incidence angle, layover will occur [50], which makes the deformation direction of the SAR imagery opposite to the actual movement direction of the glacier [51]. Therefore, the deformation pixels with a slope larger than 45° were removed, and these missing pixels were interpolated using the values of the closest deformation pixels. After these steps, the deformation direction of the SAR imagery was in accordance with the actual movement direction of the glacier. Glacier deformation can be interpreted as follows: negative deformation includes glacier ablation, down-slope movement, and thickness decrease, while positive deformation includes glacier accumulation, upslope movement, and thickness increase. For the Sentinel-1A SAR sensor, these interpretations can be explained by Figure 2, and the profile along the slope of the glacier central line (i.e., the line of symmetry of the glacier) was designed. As the glacier is symmetrical along the central line, deformations in the north and south directions can offset each other. Another reason to ignore the deformations of the north and south directions is that the LOS is not the line of symmetry of the north and south directions, and introducing deformation in the north and south directions would lead to unnecessary errors. Based on the above assumptions, the glacier movement projected on the LOS consists of two main parts: (1) along-slope movement and (2) thickness variation which is perpendicular to the horizontal line, or along the zenith. The LOS is perpendicular to the horizontal line, while the slope direction is from top to bottom. The solid angle θ (shown in Figure 2) [52] between the horizontal line and the slope is negative. This means that the deformation along slope projected on the LOS is negative and deformation in the direction of thickness variation is negative since these are both moving away from the satellite along the LOS.

The data were analyzed using the ENVI 5.3 and SARSCAPE 5.2.1 software, and the image parameters are listed in Table 2. We processed a set of 19 SAR images that were acquired by Sentinel-1A sensors between November 2017 and December 2018. All of the Sentinel-1A images used in this study were VV polarized with ascending orbit and were acquired during both the melting and refreezing seasons of the glaciers in the study region. The SRTM DEM of the study area and precise orbital information were used for the generation of interferograms [42].

**Figure 3:**
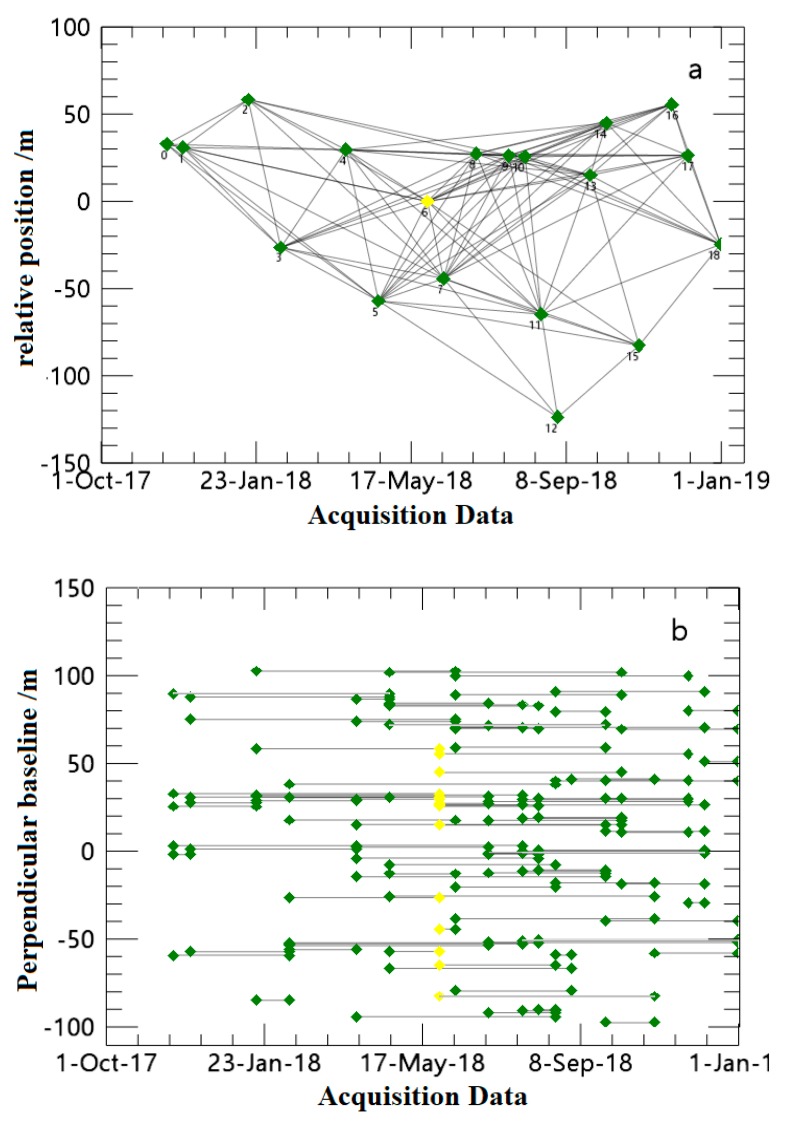
Spatial and temporal baselines of the Sentinel-1A data used in this study. (**a**) Spatial–baseline plot, the distance between two points represents the size of spatial baseline. (**b**) Time–baseline plot. The image pairs are connected by lines, the images are represented by green circles, and the super main images are represented by yellow circles; the distance between two points represents the size of time baseline.

The acquisition date of the super main image was 29 May 2018. The first step in the interferogram generation process was to generate a connection graph. According to the meteorological conditions and data sets of the study area, the maximum length of the spatial baseline was 102 m, the minimum length of the spatial baseline was 3 m, the maximum length of the temporal baseline was 192 days, and the minimum length of the temporal baseline was 12 days. The distribution of small baseline pairs is shown in Figure 3. From the figure, it can be seen that a connection is established between each time phase and other time phases and there are no discarded images.

**Figure 4:**
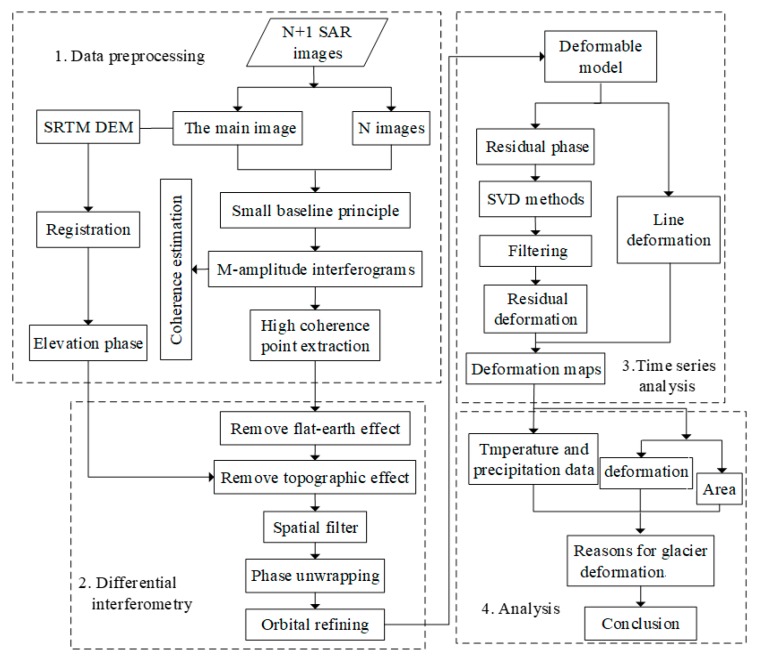
Flowchart showing the processing steps of the Small Baseline Subsets (SBAS) Interferometric Synthetic-Aperture Radar (InSAR) method used in this study. Singular Value Decomposition (SVD) is a method for mosaicking interferograms.

The most critical step in the interferometric process is the interferogram generation. The interference pairs were screened to eliminate the irregular interference fringe and unsatisfactory phase unwrapping. Finally, 87 interference pairs were reserved for use in the subsequent calculation to ensure sufficient redundant observations in the time series calculation. In order to reduce the influence of noise in the coherent region, the multiple look operation is carried out, and the number of views is 1 (azimuth direction) × 5 (distance direction) [42]. Next, a Goldstein filter and the Minimum Cost Flow (MCF) method for unwrapping were used to improve the effect and quality of the interference and phase unwrapping [53]. The processing steps of the SBAS InSAR method are shown in Figure 4.

## 3. Results

**Figure 5:**
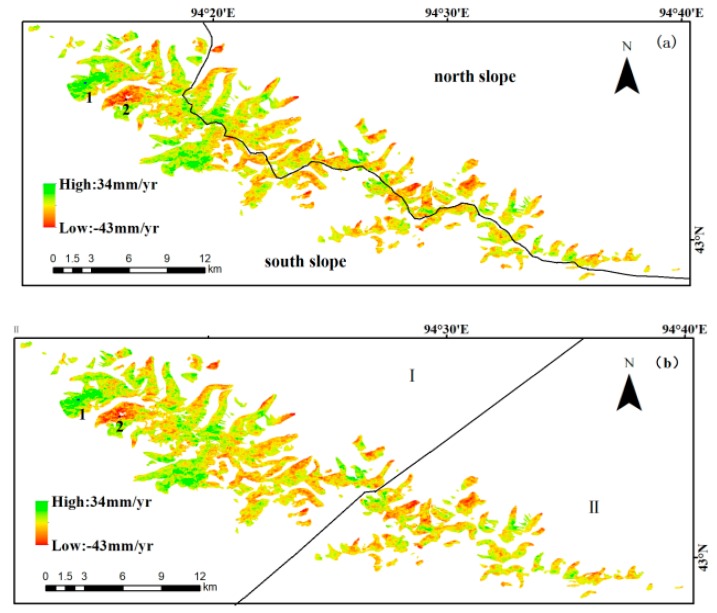
Average annual change rate of glacier deformation obtained from the SBAS InSAR analysis. (**a**) Deformation of north and south slopes. (**b**) Deformation of large glaciers (Area I) and smaller glaciers (Area II).

According to the previously described method, using glacier vector boundaries of the second China Glacier Inventory (CGI2), the average rate of glacier deformation on Karlik Mountain during the period from November 2017 to December 2018 was finally obtained (Figure 5). In Figure 5, the positive values mean that the deformation is toward the satellite along the LOS, and the negative values mean that the deformation is away from the satellite along the LOS. The maximum deformation rate of the glaciers towards the LOS is 34 mm/year, as shown by Point 1 in Figure 5, while the maximum deformation rate of the glaciers away from the satellite is 43 mm/year, as shown by Point 2 in Figure 5.

Based on the topography, the study region was divided into north and south slopes by the ridge line (Figure 5a). Due to the severe influence of the westerly wind in this area, Karlik Mountain has a northwest–southeast orientation. In this paper, the perpendicular of mountain direction was used to separate the mountain into two parts, with Area I as northwest and Area II as southeast (Figure 5b). The average rate of deformation was calculated for the north and south slopes, respectively, and the results are listed in Table 3 As shown in the table, the average glacier deformation velocity on the south slopes is 13.15 ± 8.89 mm/year and that on the north slopes is 12.38 ± 7.46 mm/year, with both deformations being away from the satellite along the LOS. Moving away from the satellite along the LOS in part represents glacier melting, while moving towards the satellite along the LOS in part represents glacier accumulation. This conclusion is consistent with the observation that the glacier deformation velocity on the south and north slopes of Karlik Mountain were more different after 1999, and the ice loss on the south slopes was greater than that on the north slopes. As shown in Figure 5b, the larger glaciers are taken as Area I and the smaller glaciers are taken as Area II. As shown in Table 3, the average glacier deformation velocity of Area I is −9.45 ± 7.65 mm/year and that of Area II is −12.16 ± 8.70 mm/year, with both deformations being away from the satellite along the LOS. This observation suggests that the deformation rates of smaller glaciers are larger than those of larger glaciers, which in turn suggests that smaller glaciers are more sensitive to climate change.

**Figure 6:**
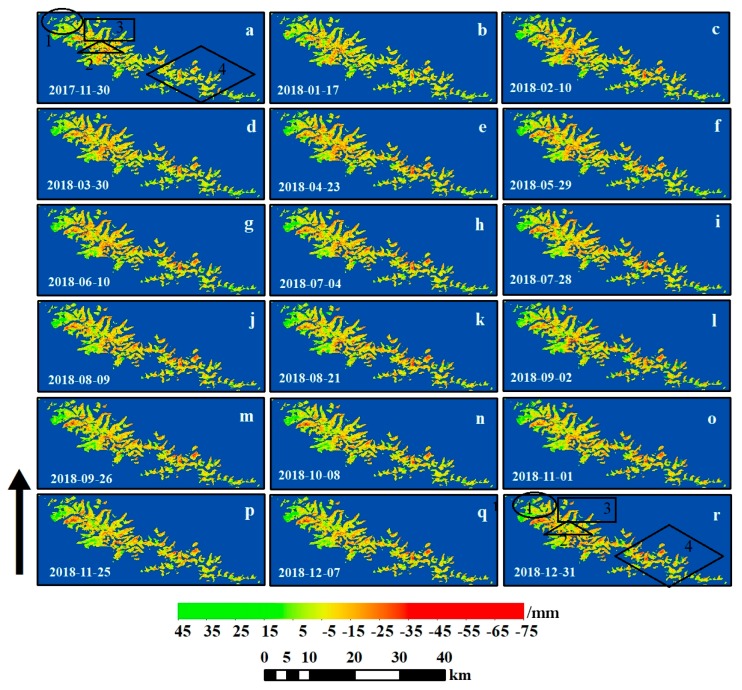
(**a**–**r**) Time series of calculated glacier deformations relative to 18 November 2017 from 30 November 2017 to 31 December 2018. Positive and negative values represent movement towards and away from the satellite along the LOS, respectively. The black arrow represents the LOS direction, which is from satellite to the ground, meanwhile from south to north.

According to the observed glacier deformation, a deformation time series was established, as shown in Figure 6. The four black polygons in Figure 6a,r denote Regions 1–4. From Figure 6a,r it can be seen that in Region 1 (far northwest of the study area), glaciers are more spatially concentrated and larger in area. Over the study period, the sign of the deformation in this region is mainly positive and its absolute value is high. In Regions 2 and 3 (northwest of the study area), the deformation of central glaciers near the ridge line is mainly positive, however, the deformation at the terminus, which is mostly composed of clean ice, is negative. This is due to the fact that glaciers tend to extend to the lower thickness of the region, the altitude of the glacial terminus is relatively low, and the glaciers terminus therefore shows a strong melting characteristic with thickness decrease and movement downslope. A total of 60% of the glaciers smaller than 1 km^2^ are located in Region 4 (southeast of the study area), with a relatively discrete spatial distribution. The deformation of glaciers in this region is mostly negative during the study period. The glacier of Region 4 is a small area with short length, the deformation contributed by the slope direct would be smaller, and the contribution by the thickness variation would be larger. Region 4 is more affected by high temperature in summer, and the deformation is therefore strongly negative. The above analysis shows that the retreat rate of glacier is slowest in the northwest and fastest in the southeast [54]. Additionally, regarding glacier deformation, the deformation of larger glaciers is mainly positive, while that of smaller glaciers is mainly negative. This phenomenon is consistent with the previous finding that small glaciers are more sensitive to climate change [55].

During the study period from 18 November 2017 to 31 December 2018, the average deformation of the glacier area was −11.77 ± 9.73 mm/year, and the total deformation to the north and south of the ridge line was −11.31 ± 6.54 mm/year and −12.11 ± 8.22 mm/year, respectively. Yang et al. [56] showed that the average thickness of the glacier on Karlik Mountain decreased by 2 mm/year between 1972 and 1999, which is a smaller decrease rate than that observed in this study. Yang et al. [56] also found that the average thickness of the glacier varied by −42 mm/year from 1999 to 2016. From 1972 to 1999, the glacier thickness decreased by 2 mm/year; from 1999 to 2016, the thickness variation increased to −42 mm/year [56]. In the present study, the glacier deformation from 2017 to 2018 was slower (−11.77 mm/year). One reason for this is that in this study deformation included not only thickness variation but also along-slope movement. The results of this study indicate that deformation due to along-slope movement was much larger than the thickness variation. Furthermore, since temperature and precipitation are the two main causes of glacier changes, the differences between the findings of the present study and the study of Yang et al. could be due to differences in these factors. Additionally, as the result of Yang X et al. [56] is an average over 27 years, it may include many extreme values. However, despite this, the overall characteristics of the results obtained in the present study are similar to those obtained by Yang et al. [56].

## 4. Discussion

### 4.1. Analysis of the Deformation Results

**Figure 7:**
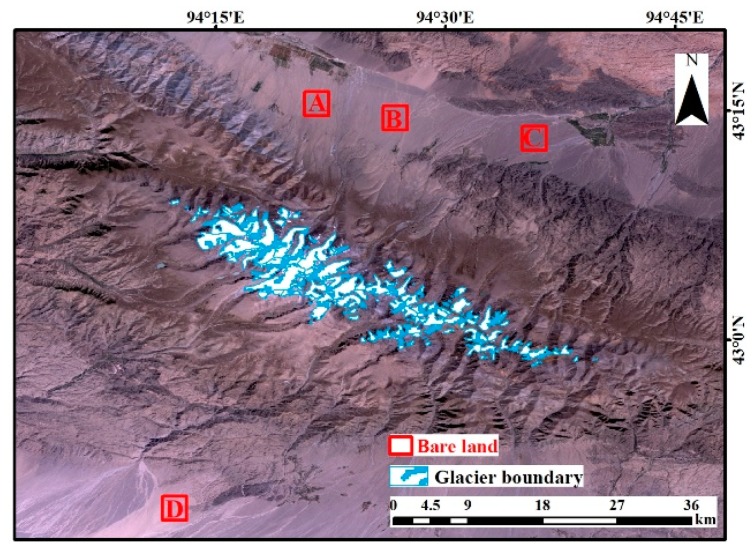
The bare land regions which were used to verify the glacier deformation results.

Deformation monitored using the SBAS InSAR method is generally verified by field observation data obtained by leveling or Global Positioning Systems [57]. However, due to the extreme elevation and climatic conditions of the present study area, it was not possible to transport personnel to collect such field observation data for reference. Therefore, to verify the reliability of the deformation monitoring results, we compared the deformation of bare land and glaciers. There is a large amount of bare land (i.e., land which has no vegetation cover all year round) around the study are, and the surface deformation of this land is stable, that is, the deformation value can be considered to be zero [58,59,60,61]. Therefore, in this study, the average deformation of bare land was taken as the error value which is caused by the systematic errors coming from satellite and the observation environments. The bare land deformation was taken as the observation value in order to evaluate the accuracy of the glacier deformation. Four bare land regions were selected to calculate the average deformation in the area, as indicated by the red squares (A, B, C, and D) in Figure 7. The calculated results are shown in Table 4. According to the analysis in Section 3, the average glacier deformation is 11.77 mm. As shown in Table 4, the deformation in the four bare land regions is 0.93, 0.63, 0.86, and 0.78 mm, respectively, accounting for only 7.9%, 5.3%, 7.3%, and 6.6% of the glacier deformation. That is, the error value is much smaller than the observed deformation value, and the reliability of the SBAS deformation monitoring results was therefore verified.

### 4.2. Effects of Climate Change on Glacier Deformation

In the study area, there is relatively more precipitation in summer due to the monsoon of the westerlies, which would bring warm humid air to the study area in summer, while there is less precipitation in winter due to the high pressure of the Inner Mongolian Siberian [32]. According to observational data from the Yiwu meteorological station (located on the northeast of Karlik Mountain), the precipitation from May to September accounted for 79.6% of the total annual precipitation, while winter precipitation accounted for only 8%.

**Figure 8:**
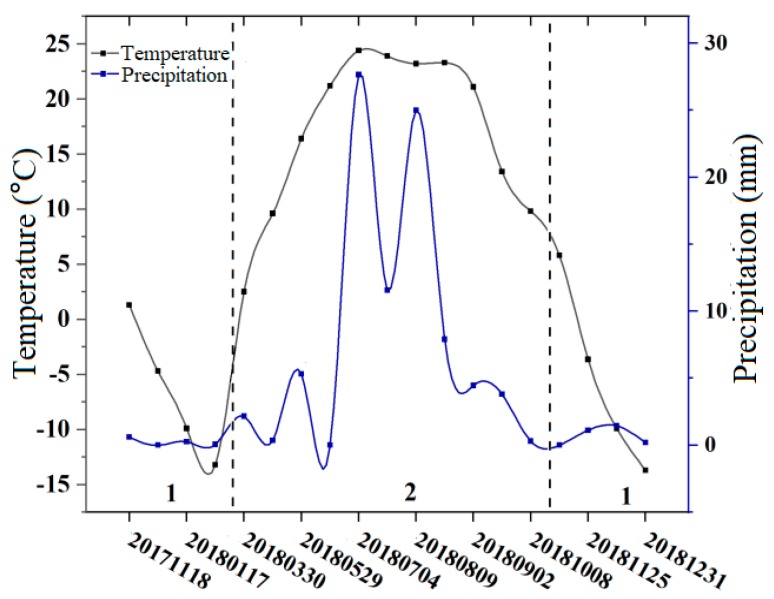
Temperature and precipitation in the study area. Period 1 refers to winter and Period 2 refers to summer.

**Figure 9:**
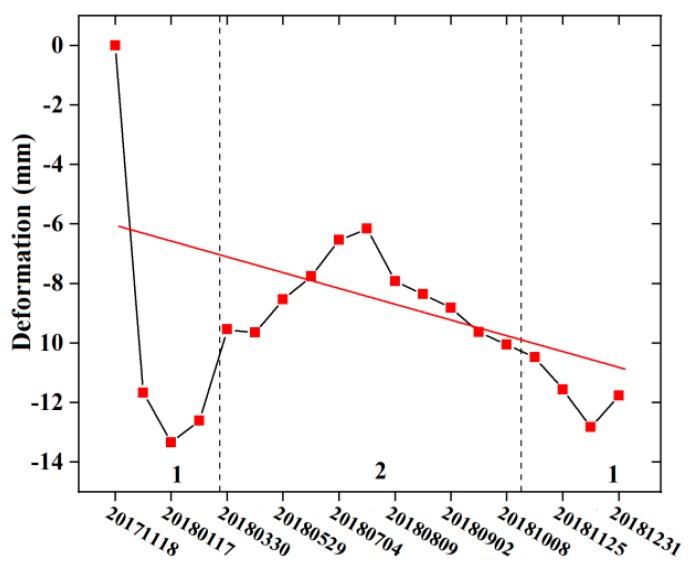
Trend of glacier deformation in the study area. Period 1 refers to winter and Period 2 refers to summer.

Data from the Yiwu meteorological station and the Hami meteorological station (located to the southwest of Karlik Mountain) (Figure 8) were used to analyze the influence of precipitation and temperature on glacier deformation (Figure 9). The original meteorological data are daily data, however in order to match the time period of glacier deformation, daily temperature and precipitation data from these two meteorological stations were averaged for winter and summer, respectively. The temperature and precipitation for these two periods are shown in Figure 8 and the glacier deformation for these two periods is shown in Figure 9.

The results show that the glaciers of Karlik Mountain were replenished in summer. From Figure 8, it can be seen that there was more precipitation in summer, which is the main reason for the lower variation of glacier deformation in summer (Figure 9). The linear fitting of the trend of glacier deformation shown in Figure 9 shows that the overall glacier deformation in the study region exhibited a decreasing trend from 2017 to 2018. In winter, the temperature was low, with a small amount of liquid precipitation and a large amount of solid precipitation [54]. No significant changes in glacier deformation were observed for this period. Meanwhile, in summer, the temperature gradually began to increase and the precipitation increased [35,39]. During this period, the temperature is higher and the liquid precipitation in the glacier area increases, thus accelerating glacier melting. On the other hand, the abundant precipitation cools down at night and freezes [35,39]. Therefore, the glacier deformation changed greatly in summer.

The above changes in glacier deformation are roughly consistent with the annual changes in the glacier movement on Karlik Mountain. Qin et al. [54] note that October to March is the accumulation period for glaciers on Karlik Mountain, more accumulation occurs in April to June than in the melting period (July to August), and the least melting occurs in September [62]. The reason why the results of the present study are not completely consistent with those of Qin et al. may be that the study periods of the two works are very different, meaning that the precipitation and temperature may also be different.

**Figure 10:**
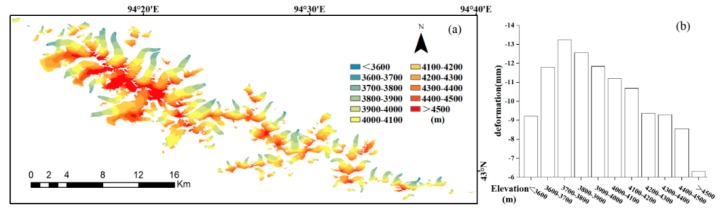
(**a**) Glacier elevation (m) in the study area. (**b**) Statistics of measured glacier deformation at different elevations.

Glacier deformation is closely related to glacier retreat and is strongly affected by elevation. In this study, glacier elevation was divided into 11 different altitude bands (Figure 10a) to investigate the relationship between glacier deformation and elevation (Figure 10b). A comparison between Figure 10a,b shows that the absolute value of deformation is high for glacier elevations of [3600, 3800] m. This can be attributed to the fact that these relatively low elevations are exposed to higher temperatures. However, within this interval, the absolute value of deformation shows an upward trend with increasing altitude, reaching a maximum value near [3700, 3800] m. This can be attributed to the fact that the main distribution area of the ice tongue—which is covered with debris—occurs at elevations lower than 3700 m [11,12,62]. Debris cover can inhibit melting. Although the altitude of the ice tongue is relatively low, the absolute value of deformation is small. Meanwhile, glaciers in the altitude range of [3800, 4200] m are distributed on both sides of the ridgeline. The absolute value of deformation in this range is lower than that in the range [3600, 3800] m. With a decreasing distance to the ridge line, the altitude increases and the absolute value of deformation decreases gradually. At altitudes above 4500 m, there is almost no deformation. This observation is consistent with the conclusion that the retreat rate of the glaciers of Karlik Mountain between 1973 and 2016 showed a decreasing tendency with increasing altitude. Below 4200 m, the deformation rate of the glaciers varied significantly, while above 4500 m, it was relatively stable [54].

## 5. Conclusions

In this paper, the SBAS InSAR method was used to monitor the variation of glaciers in the Eastern Tienshan Mountains between November 2017 and December 2018. The glacier deformation rate was calculated and a deformation time series was presented. The main conclusions are as follows:

(1) A SAR dataset with a short temporal baseline and a short spatial baseline was established. By setting appropriate temporal and spatial baseline thresholds, incoherence effects caused by long spatial and temporal baselines can be limited. Thus, the glacier deformation accuracy was improved;
(2) The total glacier deformation during the study period was −11.77 ± 9.73 mm;
(3) Based on the assumption that the deformation of bare land in the study area is zero, the deformation of bare land was taken as the error value in order to validate the deformation results. It was found that this error value is much smaller than the glacier deformation, which thus verifies the reliability of the results of this paper;
(4) Taking the ridge line as the dividing line, it was found that the deformation rate of the south slopes was higher than that of the north slopes. It was found that the deformation rate was higher in the southeast of the study area than in the northwest;
(5) Through the analysis of temperature and precipitation data, it was found that precipitation played a leading role in the glacier deformation. In winter, precipitation and temperature were low, and glacier deformation was also low. There was a strong correlation between glacier deformation and elevation; the lower the elevation, the larger the deformation.

## Figures and Tables

**Table 1:** Parameters of the Sentinel-1A sensor images and the Shuttle Radar Topographic Mission (SRTM) Digital Elevation Model (DEM) data used in this study.

Sentinel-1A Sensor Image		SRTM DEM	
Parameter	Type	Parameter	Type
Polarization mode	VV	Spatial resolution	90 m
Swath mode	IW	Data format	TIF
Data type	SLC	Reference surface	WGS-84
Spatial resolution	5 m (range) × 20 m (azimuth)	Relative elevation accuracy	±10 m
Revisit frequency Orbit type	12 Days Ascending	Absolute elevation accuracy	±16 m
Data coverage	250 km		

Note: VV: vertical transmit and vertical receive; IW: Interferometric Wide; SLC: Single Look Complex, the symbol “×”is multiplication sign.

**Table 2:** Parameters of the 19 Sentinel-1A images used in this study.

Image Number	Acquisition Date	Orbit Type	Orbit Number	Polarization
0	18 November 2017	ASCENDING	019319	VV
1	30 November 2017	ASCENDING	019494	VV
2	17 January 2018	ASCENDING	020194	VV
3	10 February 2018	ASCENDING	020544	VV
4	30 March 2018	ASCENDING	024897	VV
5	23 April 2018	ASCENDING	025380	VV
6	29 May 2018	ASCENDING	022119	VV
7	10 June 2018	ASCENDING	022294	VV
8	4 July 2018	ASCENDING	022644	VV
9	28 July 2018	ASCENDING	022994	VV
10	9 August 2018	ASCENDING	023169	VV
11	21 August 2018	ASCENDING	023344	VV
12	2 September 2018	ASCENDING	023519	VV
13	26 September 2018	ASCENDING	023869	VV
14	8 October 2018	ASCENDING	024044	VV
15	1 November 2018	ASCENDING	024394	VV
16	25 November 2018	ASCENDING	024744	VV
17	7 December 2018	ASCENDING	024919	VV
18	31 December 2018	ASCENDING	025269	VV

**Table 3:** Average glacier deformation velocity in different glacial partitions.

Glacial Partition	Deformation Velocity (mm/year)
South slopes	−13.15 ± 8.89
North slopes	−12.38 ± 7.46
Area I	−9.45 ± 7.65
Area II	−12.16 ± 8.70

**Table 4:** Deformations of the bare land regions shown in Figure 7 between 18 November 2017 and 31 December 2018.

Region	Deformation (mm)
A	0.93
B	0.63
C	0.86
D	0.78
